# Microplastics–Pollutant Interactions in Environmental Systems: Mechanisms, Ecological Effects, and Implications for Sustainable Management

**DOI:** 10.3390/molecules31111852

**Published:** 2026-05-28

**Authors:** Lei Wu, Xuerong Zhou, Cui Lai, Mingyang Ma, Lei Qin, Wenjun Wang

**Affiliations:** 1 College of Environmental Science and Engineering, Hunan University, Changsha 410082, China; s2303w0532@hnu.edu.cn (L.W.); laicui@hnu.edu.cn (C.L.); mqmingy@hnu.edu.cn (M.M.); 2 Key Laboratory of Environmental Biology and Pollution Control, Hunan University, Changsha 410082, China; 3 Changjiang River Scientific Research Institute, Changjiang Water Resources Commission, Wuhan 430010, China; 4 School of Resources and Environment, Hunan University of Technology and Business, Changsha 410205, China

**Keywords:** microplastics, co-contaminants, interaction mechanisms, environmental systems, ecological risk, sustainable management

## Abstract

Microplastics (MPs) are persistent contaminants widely distributed across aquatic, terrestrial, and atmospheric environments. Previous reviews have shown that MPs can carry pollutants and change their environmental behavior, but the field is now broad enough that a simple repetition of sources, adsorption mechanisms, and toxicity would add limited value. This review therefore organizes MPs–pollutant–interactions as a connected chain from sources and environmental pathways to interaction mechanisms, biological effects, and management actions. It summarizes the major MPs input pathways and representative polymer types across water, soil, and air, and then explains how partitioning, surface adsorption, desorption, and pore filling control the binding and release of pollutants. The review further discusses how MPs properties, pollutant characteristics, pollutant mixtures, biofilms/plastisphere, and environmental factors jointly regulate these processes. In addition, it evaluates the consequences of MPs-pollutant coupling for pollutant mobility, bioavailability, biodegradation, bioaccumulation, trophic transfer, ecological toxicity, and human exposure. Finally, the review links these processes with practical management needs, including wastewater treatment, sludge reuse, agricultural plastic control, atmospheric monitoring, environmental education, and long-term risk assessment. By bringing these topics into one framework, this review provides a clearer basis for understanding and managing MPs-associated mixed pollution in environmental systems.

## Simple Summary

Microplastics are now found in water, soil, and air, where they can interact with antibiotics, heavy metals, persistent organic pollutants, and other contaminants. This review explains how microplastics bind, release, and transport pollutants, how these processes affect environmental fate and biological risks, and why monitoring and management should treat microplastics as part of broader mixed-pollution systems rather than as isolated particles.

## 1. Introduction

The widespread production and use of plastics have brought major societal benefits, but have also created a persistent waste management challenge that increasingly threatens environmental sustainability. Owing to their durability, light weight, low cost, and versatility, plastic materials are extensively used in packaging, agriculture, textiles, personal care products, and industrial applications [1,2]. However, behind the prosperity of this “plastic age” lies a significant contradiction between the rapidly growing production of plastics and a fragile waste management system, leading to the release of vast amounts of plastic waste into the natural environment and posing a severe global environmental problem [3]. These plastic wastes do not remain intact in the environment; instead, they undergo fragmentation and aging under a series of complex environmental stresses, including UV radiation, physical abrasion, chemical degradation, and biological activity, ultimately forming an immense number of smaller plastic particles [4]. Those plastic fragments smaller than 5 mm in size are universally defined by the scientific community as MPs [5]. Recognized for their large specific surface area, persistence, and potential ecotoxicity, MPs have emerged as a globally concerning class of emerging pollutants [6].

Based on their origin, MPs are generally classified as primary or secondary particles [7]. Primary MPs are intentionally manufactured at small sizes and may be released from industrial resin pellets, personal care products, cleaning products, or other engineered materials [8]. Secondary MPs are generated through fragmentation and weathering of larger plastics such as bags, bottles, fishing nets, textile fibers, tire particles, and agricultural films [9,10]. They have been detected in marine and freshwater systems, sediments, agricultural soils, polar regions, urban and remote air, and biological tissues [5,6,11,12]. Therefore, MP pollution is not limited to a single environmental compartment. It is linked to wastewater management, sludge reuse, agricultural production, atmospheric transport, food-chain safety, and long-term circular-materials governance.

The environmental risk of MPs extends far beyond their physical presence. Owing to the characteristics of strong hydrophobicity, large specific surface area, and surfaces often carrying negative charges or specific functional groups, MPs can efficiently adsorb various co-existing inherent pollutants in the environment, including persistent organic pollutants (POPs), heavy metals, antibiotics, and personal care products [13,14,15,16]. In this sense, MPs should be viewed not only as emerging contaminants, but also as components of a coupled pollution system involving both particulate carriers and sorbed chemicals. This issue is particularly important from the perspective of sustainable environmental systems. MPs are introduced into water, soil, and air through multiple anthropogenic pathways, including wastewater discharge, sewage sludge application, agricultural plastic mulching, landfill leachate, urban dust, and atmospheric deposition [17]. Therefore, the environmental significance of MPs extends beyond their occurrence and toxicity, and increasingly intersects with broader questions of sustainable resource use, waste reuse, and long-term ecosystem protection.

Studies have indicated that the interaction mechanisms between MPs and these pollutants are highly complex, primarily involving partition dominated by hydrophobic effects, surface adsorption involving electrostatic interactions, hydrogen bonding, π–π interactions, and van der Waals forces, as well as pore-filling mechanisms dependent on the particle’s pore structure [18,19]. This interaction effect significantly alters the transport, transformation, fate, and bioavailability of pollutants. For instance, MPs can act as “Trojan horses,” transporting high concentrations of sorbed pollutants to previously uncontaminated areas, or, upon ingestion by organisms, desorb and release these pollutants under specific physiological conditions, leading to localized high-concentration exposure and resulting in combined toxic effects not seen with individual pollutants alone [13,20]. This synergistic effect can be transferred and amplified through the food chain, ultimately posing potential, long-term, and complex threats to ecosystem function and human health [21].

Although many studies have clarified the occurrence, sorption behavior, and toxicity of MPs, several issues remain insufficiently integrated. First, the physicochemical properties of MPs (polymer type, particle size, morphology, surface charge, and aging state), pollutant characteristics (hydrophobicity, polarity, charge, molecular size, and ionization state), and environmental conditions (pH, temperature, salinity, dissolved organic matter, and biological activity) jointly form a coupled regulatory network [16,18,21]. Second, laboratory adsorption–desorption data are often generated under simplified conditions, whereas real environments contain changing hydrodynamics, mineral surfaces, natural organic matter, biofilms, and mixed contaminants. Third, the atmospheric compartment has received much less attention than aquatic and soil systems, even though airborne MPs may undergo oxidative aging, long-range transport, dry/wet deposition, and inhalation exposure. These limitations indicate that a matrix-specific and mechanism-based synthesis is still needed.

Several recent reviews have already made important contributions to this field. Menendez-Pedriza and Jaumot reviewed sorption factors, bioaccumulation scenarios, and ecotoxicological effects of MPs with associated chemicals [22]. Xiang et al. summarized MP sources, transport, fate, interaction mechanisms, and toxicology, with much of the discussion centered on aquatic and soil environments [23]. Mousazadehgavan discussed aquatic MPs from the perspective of their dual role as pollutant carriers and potential functional materials for water treatment [24]. Carvalho et al. placed more emphasis on micro/nanoplastics and related chemicals in the environment, food, and human-health contexts [25]. These studies provide an important basis for the present review. However, they also show that the field now needs not only more lists of sources, mechanisms, or toxic effects, but also a clearer connection among these topics.

Therefore, this review does not claim its novelty from being the first to discuss MPs as pollutant carriers, nor only from mentioning water, soil, and air together. Instead, its contribution is to organize the evidence into a more complete and easier-to-follow chain: MP sources and environmental pathways, interaction mechanisms with pollutants, effects of pollutant mixtures and biofilms/plastisphere, ecological and human-health consequences, and management points. By doing so, the review aims to help readers move from separate observations of occurrence, adsorption, or toxicity toward a more practical understanding of how MP-associated mixed pollution should be monitored and managed.

## 2. Review Methodology

This article was prepared as a critical narrative review rather than a formal PRISMA-style systematic review or meta-analysis. The literature search was conducted in Web of Science, Scopus, PubMed, ScienceDirect, ACS Publications, SpringerLink, Wiley Online Library, and Google Scholar. The search covered publications from 2010 to 2026, with emphasis on studies published since 2020 because the number of studies on MPs–pollutant interactions has increased rapidly in the last five years.

The main search terms included combinations of the following keywords: “microplastics”, “nanoplastics”, “pollutant adsorption”, “desorption”, “partitioning”, “pore filling”, “biofilm”, “plastisphere”, “organic pollutants”, “heavy metals”, “antibiotics”, “pesticides”, “environmental fate”, “toxicity”, “human health”, “soil”, “freshwater”, “marine”, and “atmospheric microplastics”. Reference lists of highly relevant review articles and primary research papers were also screened to identify additional studies.

Studies were included when they directly addressed sources, environmental transport, physicochemical interactions, sorption/desorption mechanisms, biological effects, or management implications of MPs or MNPs. Studies were excluded when they focused only on bulk plastic waste without microplastic-scale evidence, lacked mechanistic relevance to pollutant interactions, or did not provide sufficient methodological information. Because the objective was to integrate evidence across aquatic, terrestrial, and atmospheric systems, both experimental and review studies were considered, while greater weight was given to peer-reviewed studies with clear environmental relevance.

The results and discussion below are organized to follow this review logic: sources and environmental pathways of MPs, mechanisms of interaction with pollutants, controlling factors, ecological and health effects, and implications for sustainable management.

## 3. Results and Discussion

### 3.1. Sources and Environmental Pathways of MPs

**Figure 1:**
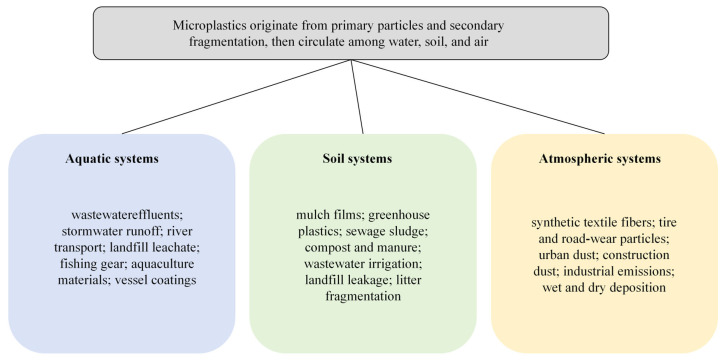
Putative sources and environmental pathways of MPs in aquatic, soil, and atmospheric systems.

MPs originate from both intentionally manufactured primary particles and secondary particles produced by fragmentation, weathering, abrasion, and aging of larger plastic products [26]. Their sources differ substantially among environmental compartments; therefore, a source-oriented discussion is more informative than a general list of plastic products (Figure 1). In aquatic systems, major entries include wastewater effluents, stormwater runoff, riverine transport, landfill leachate, fishing and aquaculture debris, and atmospheric deposition. In soils, the dominant putative sources include agricultural plastic films, greenhouse materials, sewage sludge, compost or manure amendments, wastewater irrigation, landfill leakage, and litter fragmentation. In the atmosphere, MPs are mainly released as textile fibers, tire and road-wear particles, construction dust, urban dust, and particles resuspended from contaminated surfaces. These sources are connected through runoff, deposition, resuspension, and biological transport, indicating that MPs circulate among water, soil, and air rather than remaining in one compartment.

In aquatic environments, MP inputs can be divided into marine-based and land-based sources. Marine-based sources include abandoned or lost fishing gear, aquaculture floats and nets, vessel coatings, shipping-related resin pellets, and plastic debris from offshore activities [27]. Land-based sources are usually more diverse and include domestic and industrial wastewater, stormwater runoff, river transport, landfill leachate, and improperly managed plastic wastes [8,28]. Laundry-derived synthetic fibers, personal care product particles, and fragments from wet wipes and sanitary materials can enter sewer systems and then pass through or accumulate in wastewater treatment plants [28,29]. Rivers and drainage networks subsequently transport these particles to lakes, estuaries, and coastal waters. Once in aquatic systems, MPs may remain suspended, settle into sediments, strand on beaches, or be ingested by organisms, thereby creating additional biological transport pathways [30,31].

Freshwater and marine systems also differ in transport processes. In rivers, hydrodynamic flow, seasonal discharge, wastewater inputs, and urban runoff strongly influence MP abundance and movement. In estuaries and coastal waters, salinity gradients, aggregation with organic matter, biofouling, and density changes determine whether MPs remain in the water column or sink to sediments. These processes are important for pollutant interactions because the same MP particle may experience changes in ionic strength, dissolved organic matter, and attached biofilms during transport, which can modify adsorption and desorption behavior.

In soil environments, agricultural activity represents one of the most important sources of MPs. Plastic mulch films and greenhouse covers undergo photo-oxidation, mechanical fragmentation, and tillage-induced abrasion, gradually producing MPs that can accumulate in topsoil [17]. Sewage sludge and compost application are also important pathways because wastewater treatment retains a large proportion of influent MPs in sludge rather than removing them completely from the waste stream [32]. When sludge is reused as fertilizer, MPs and associated contaminants may be transferred to agricultural soils; model estimates and field investigations have shown that sludge application can substantially increase MP inputs to farmland [33,34]. Other soil sources include wastewater irrigation, livestock manure, landfill leachate, road runoff, construction residues, and litter fragmentation.

The sources of MPs in soils should also be linked to their putative source materials. For example, polyethylene and polypropylene are often associated with mulch films, packaging, and disposable plastics; polyester and polyamide fibers are frequently related to textiles and sludge; tire-wear particles and rubber fragments are associated with roads and urban runoff; and polystyrene or polyurethane fragments may originate from packaging, insulation, and foam materials. Such source identification is important because polymer type, additive composition, crystallinity, and aging state strongly affect the capacity of MPs to bind antibiotics, persistent organic pollutants, and metals.

Compared with aquatic and soil systems, atmospheric MPs remain less studied but are increasingly recognized as an important transport and exposure pathway. Indoor air is often enriched in fibers released from synthetic textiles, carpets, furniture, and household dust, whereas outdoor air can contain tire-wear particles, road dust, construction debris, industrial emissions, landfill- or incineration-related particles, and resuspended soil or urban dust [35,36,37,38,39]. Airborne MPs can undergo photo-oxidation and physical fragmentation during transport, and then return to aquatic and terrestrial systems through wet and dry deposition. Therefore, the atmospheric compartment should not be treated only as an exposure route for inhalation; it is also a pathway connecting urban sources with remote aquatic and soil ecosystems.

### 3.2. Mechanisms of Interaction Between MPs and Pollutants

The interactions between MPs and environmental pollutants involve coupled physical, chemical, and biological processes. The dominant mechanism depends on the environmental matrix, polymer type, particle size and aging state, pollutant chemistry, and surrounding conditions. In aquatic systems, hydrophobic partitioning, surface adsorption, salinity-dependent aggregation, and DOM competition are often important. In soils, mineral surfaces, soil organic matter, pore structure, moisture, and microbial biofilms may regulate pollutant binding and mobility. In the atmosphere, MPs interact with hydrophobic organic chemicals, metals, and secondary aerosols during airborne transport, while UV exposure and oxidants can alter their surface chemistry before deposition. The following sections therefore describe the major mechanisms and emphasize how they may differ across water, soil, and air.

#### 3.2.1. Adsorption

In general, the adsorption mechanisms of pollutants on MPs typically include partitioning effects, surface adsorption, and other mechanisms, with partitioning and surface adsorption being the two primary mechanism [15,40,41,42].

##### Influence of Partitioning

Partitioning is a key mechanism for pollutant adsorption onto or within microplastic surfaces. Its core principle involves the dynamic equilibrium of pollutant distribution between MPs (acting as a pseudo-organic phase) and the environmental medium (e.g., aqueous phase). The driving force behind partitioning is pollutant hydrophobicity, independent of the number of surface physical adsorption sites. Its influencing factors depend on polymer properties, pollutant hydrophobicity, and environmental conditions.

MPs are hydrophobic in nature and form a partition equilibrium with hydrophobic pollutants through hydrophobic interactions. The classical model for this process is typically based on linear partition theory, where the adsorption capacity of pollutants is positively correlated with their concentration in the aqueous phase. Experimental studies have verified that the adsorption behavior of pollutants onto MPs conforms to the linear partition model. For example, Xu et al. found that the adsorption of polyethylene(PE) onto sulfamethoxazole could be fitted to a pseudo-second-order model (R^2^ = 0.98) and a linear model (R^2^ = 0.99), indicating that the adsorption process is primarily influenced by distribution effects [43]. Similarly, Wang investigated the adsorption process of perfluorooctane sulfonate (PFOS) and perfluorooctane sulfonamide (FOSA) onto MPs [41]. The adsorption isotherms exhibited linear behavior, indicating that the pollutants were adsorbed onto the MPs via the distribution mechanism.

The distribution effect is closely related to the polarity and aromaticity of MPs. The polarity index (PI) is typically expressed as (O + N)/C [44], H/C is used to indicate the degree of aromaticity in MPs and the magnitude of the equilibrium distribution coefficient (Kd) is often used to measure the adsorption capacity of MPs for organic pollutants [15,45]. As the PI value and H/C ratio of MPs decrease, their polarity diminishes and aromaticity increases, leading to a larger Kd value and stronger partitioning effect on pollutants. Conversely, as the PI value and H/C ratio increase, polarity rises and aromaticity weakens, resulting in a smaller Kd value and consequently reduced partitioning effect on pollutants [45]. Teuten et al. summarized the physicochemical mechanisms governing interactions between MPs and pollutants, noting that partitioning dominates the adsorption of nonpolar pollutants (e.g., polychlorinated biphenyls DDT) onto nonpolar MPs (e.g., polyethylene, polypropylene) [46].

Finally, environmental parameters such as temperature, salinity, and dissolved organic matter indirectly influence the strength of partitioning effects by altering pollutant solubility or microplastic surface properties. Studies have shown that increased salinity reduces the solubility of hydrocarbon organic compounds (HOCs) through a salting-out effect, thereby enhancing the partitioning of hydrophobic organic pollutants into MPs [47,48]. Also, elevated temperatures increase the kinetic energy of pollutant molecules, promoting their diffusion and permeation within MPs [49]. Dissolved Organic Matter(DOM) inhibits the partition by competing with pollutants for adsorption sites on microplastic surfaces [22].

##### Surface Adsorption

**Figure 2:**
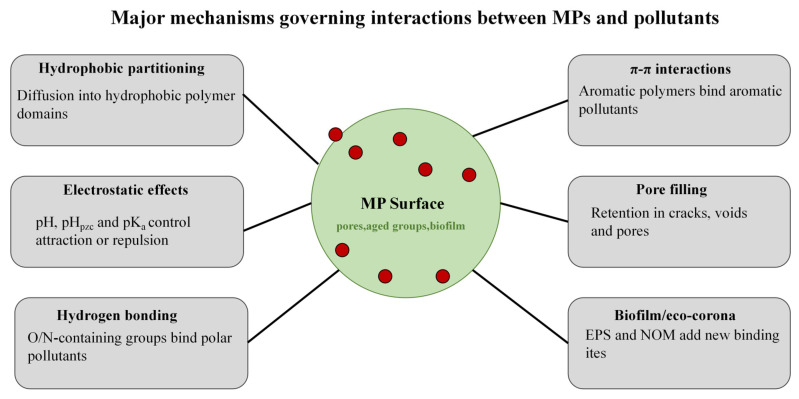
Major mechanisms governing interactions between MPs and pollutants, including hydrophobic partitioning, electrostatic interactions, hydrogen bonding, π–π interactions, pore filling, and biofilm/eco-corona effects.

Surface adsorption refers to the process by which MPs adsorb dissolved pollutants or colloids from water. This process primarily relies on intermolecular forces and involves various noncovalent interactions between the microplastic surface and pollutant molecules [50]. The mechanisms of surface adsorption primarily include hydrophobic interaction, electrostatic interaction, hydrogen bonding, π–π interactions, and van der Waals forces (Figure 2).

Due to the high hydrophobicity of MPs in the environment, hydrophobic interactions serve as the primary mechanism for microplastic adsorption of hydrophobic pollutants. These interactions essentially involve the aggregation and adsorption of pollutants onto hydrophobic surfaces within aquatic environments, driven by the mutual repulsion of water molecules. Research indicates that this effect correlates with the hydrophobicity of MPs: the stronger the hydrophobicity, the greater the adsorption of pollutants and the more pronounced the hydrophobic interactions [16,51]. For example, Guo et al. found that the adsorption capacity of tylosin (TYL) on different MPs followed the order of PE < PP < PS < PVC, while the hydrophobicity of the four MPs varied significantly. This indicates that hydrophobic interactions are related to the hydrophobicity of MPs [52]. Wu et al. found that the octanol/water partition coefficients (K_ow_) for carbamazepine (CBZ), 17α-ethynylestradiol (EE2), triclosan (TCS), and 4-methylbenzylidene camphor (4MBC) were 2.45, 3.67, 4.76, and 5.10, respectively. Their corresponding adsorption coefficients on PE were 191.4, 311.5, 5.14 × 10^3^ and 5.32 × 10^4^ L/kg, indicating a positive correlation between pollutant adsorption and microplastic hydrophobicity [53].

When MPs and pollutants both carry electrical charges, electrostatic interactions occur. This phenomenon can be understood by the principle that “like charges repel each other, while opposite charges attract”. The strength of electrostatic interactions depends on the zero charge point (pH_pzc_) of MPs and the pK_a_ (dissociation constant of the compound) of environmental pollutants relative to the solution pH [54]. When pH_pzc_ < pH, MPs carry a negative charge and readily attract positively charged pollutants; when pH_pzc_ > pH, MPs carry a positive charge and readily attract negatively charged pollutants [55]. When pH = pK_a_, organic pollutants exist in molecular form; when pH < pK_a_, organic pollutants carry a positive charge; when pH > pK_a,_ organic pollutants carry a negative charge. Therefore, whether electrostatic attraction or repulsion occurs depends on the combined effects of factors such as the medium’s pH value, the MPs’ pH_pzc_, and the organic pollutant’s pK_a_. Generally speaking, the pH_pzc_ values of most common MPs (such as PP, PS, and PE) are lower than the pH of most aquatic environments (4.26, 3.96, and 4.30, respectively). Consequently, MPs in the environment predominantly carry negative charges, making them highly susceptible to adsorbing positively charged pollutants [54]. Li et al. found that in experimental water bodies, ciprofloxacin (CIP) existed in its cationic form, while the environmental pH exceeded the pH_pzc_ of MPs, causing the MPs to carry a negative charge. Consequently, through electrostatic adsorption, the CIP cation enhanced its adsorption capacity onto the negatively charged surface of the MPs [15]. Razanajatovo et al. investigated the adsorption behavior of PE in freshwater (pH = 6.85) toward two positively charged drugs, propranolol (PRP) and sertraline (SER), and one negatively charged drug, sulfamethoxazole (SMX). With a pH_pzc_ of 4.30, PE carried a negative charge in freshwater, exhibiting electrostatic repulsion toward similarly negatively charged SMX while attracting positively charged PRP and SER. Consequently, in the isotherm linear model, SMX exhibited a lower K_d_ value (0.70 × 10^3^ L/kg) compared to SER and PRP (3.33 × 10^3^ and 2.30 × 10^3^ L/kg, respectively), indicating weaker adsorption capacity on PE than SER and PRP [56].

Moreover, hydrogen bonding and π–π interactions also play a significant role in the adsorption of pollutants by MPs. Hydrogen bonds are a significant type of intermolecular force, typically occurring between hydrogen atoms and more electronegative atoms such as oxygen and nitrogen. Their formation is related to the surface functional groups of MPs [57]. MPs surfaces may harbor polar functional groups containing oxygen or nitrogen, such as hydroxyl (-OH), carboxyl (-COOH), and amino (-NH_2_) groups. The hydrogen atoms or oxygen/nitrogen atoms within these functional groups can form hydrogen bonds with atoms in contaminant molecules that act as hydrogen bond acceptors or donors [58,59]. Kuang et al. investigated the interaction of sulfamethoxazole (SMZ) with PP, PE, PS, and PVC under varying temperature and salinity conditions. The results indicate that hydrogen bonding participates in the adsorption process of SMZ onto MPs [60].

In natural environments, MPs undergo aging processes such as photodegradation and weathering, leading to the formation of numerous oxygen-containing functional groups on their surfaces, such as -OH and -COOH groups. These functional groups contain highly electronegative oxygen atoms and reactive hydrogen atoms, enabling them to act as both hydrogen bond donors and acceptors. This enhances the hydrogen bonding interactions between MPs and pollutants [58,61]. For instance, studies have found that ultraviolet-aged PS and polybutylene adipate-co-terephthalate (PBAT) exhibit enhanced adsorption capacity for Rhodamine B (RhB). This phenomenon is attributed to charge-assisted hydrogen bonding resulting from increased surface functional groups on the MPs [58,62].

π–π interactions are noncovalent interactions that primarily occur between aromatic rings rich in π electrons, enabling intermolecular attraction through the overlap of the rings’ electron clouds [57]. π–π interactions require at least one of the microplastic and contaminant molecules to contain an aromatic ring structure. MPs such as PS and PVC possess polymer structures containing benzene rings or unsaturated π bonds. These π-electron systems can form π–π interactions with pollutant molecules possessing aromatic ring structures, such as PAHs and phenolic compounds [63,64]. For example, study on the interaction between TCS and MPs indicated that π–π interactions play a crucial role in the adsorption of TCS [65]. The strength of this effect is related to the aromaticity of MPs, their degree of aging, and the planarity of contaminant molecules.

Van der Waals forces are a class of ubiquitous weak intermolecular forces, including orientation forces, induction forces, and dispersion forces (London forces) [66]. Although van der Waals forces are weaker than chemical bonds (such as covalent bonds and ionic bonds), they still play a vital role in the physical adsorption between nonpolar or weakly polar molecules and surfaces. They are also the primary driving force for the adsorption of pollutants onto microplastic surfaces [67,68]. For example, adsorption kinetics experiments of 9-nitroanthracene on polyethylene, polypropylene, and polystyrene MPs indicate that van der Waals forces are a key mechanism governing its adsorption [69]. Additionally, Wang et al. conducted molecular dynamics simulations of tetracycline adsorption behavior in carbon nanopores, quantifying the contribution of van der Waals forces to the adsorption energy. This directly reflects the role of van der Waals forces in the adsorption process [70].

##### Effects of Pollutant Mixtures on MP Adsorption

In real environmental systems, MPs rarely encounter a single pollutant. Mixtures of organic pollutants, antibiotics, pesticides, heavy metals, nutrients, and natural organic matter can produce competitive, cooperative, or sequential adsorption behavior. Competitive adsorption occurs when compounds with similar hydrophobicity, charge, or molecular size occupy the same surface sites or pore regions on MPs. In contrast, cooperative adsorption may occur when metal ions form cation bridges between negatively charged MPs and anionic organic pollutants, or when natural organic matter forms a coating that creates additional binding domains.

Therefore, single-solute adsorption coefficients cannot always be directly extrapolated to mixed-pollutant conditions. The apparent affinity of MPs for one pollutant may decrease because of site competition, increase because of bridging or co-sorption, or change dynamically during aging and biofilm development. Future experiments should therefore include binary and multi-component systems that better represent wastewater, agricultural soil, estuarine water, and atmospheric deposition scenarios.

##### Importance of Biofilms and the Plastisphere

After entering natural environments, MPs are rapidly colonized by microorganisms and extracellular polymeric substances, forming biofilms or a plastisphere. Biofilms can modify the surface charge, hydrophobicity, roughness, and functional-group composition of MPs, thereby changing their affinity for pollutants. Extracellular polymeric substances contain carboxyl, hydroxyl, amino, and phosphoryl groups that may bind metals and polar organic pollutants, while microbial organic matter can also provide hydrophobic domains for nonpolar compounds.

The biofilm effect is therefore not unidirectional. In some cases, biofilm growth increases pollutant retention by introducing new binding sites; in other cases, it masks the original polymer surface or enhances desorption through biodegradation, enzymatic activity, or colloidal stabilization. Biofilm-mediated interactions are especially important in wastewater treatment, rivers, sediments, and agricultural soils, where MPs can act as carriers not only for chemicals but also for pathogens and antibiotic-resistance genes.

#### 3.2.2. Desorption

Desorption is the reverse process of adsorption, referring to the release of pollutants adsorbed onto the surface or interior of MPs back into the surrounding environment (such as liquids or gases). Since pollutants adsorbed onto MPs may desorb within organisms after being ingested, potentially causing toxicity, studying desorption is crucial for assessing the environmental migration, transformation, and ecological risks associated with MPs and pollutants [71,72,73]. When MPs migrate from areas with high pollutant concentrations to areas with low concentrations, pollutants desorb from the microplastic surface to achieve a new equilibrium [74]. Environmental conditions such as pH and temperature influence the surface charge of MPs and the form of contaminants, thereby affecting desorption processes. For instance, experiments on the desorption of SMX from polyamide (PA6) MPs in simulated gastrointestinal fluids systematically validated the impact of environmental conditions (pH, salinity) on the desorption process [73]. Zhang et al. conducted desorption experiments of copper (Cu(II)) from polystyrene MPs in different media (ultrapure water, artificial seawater, simulated gastric fluid) at varying temperatures. The results indicated that the desorption rate was highest in simulated gastric fluid, followed by ultrapure water and artificial seawater, and that increasing temperature enhanced desorption efficiency [75].

MPs, with their large specific surface area and strong hydrophobicity, can act as “Trojan horses”, transporting adsorbed pollutants (such as heavy metals, organic pollutants, and antibiotics) into organisms. When organisms ingest MPs containing pollutants, the pollutants adsorbed on the microplastic surface may desorb in specific environments like the digestive tract and be absorbed by the organism. This leads to increased local concentrations of pollutants within the organism, enhancing their biological toxicity [71,72,76,77,78]. Bisphenol A (BPA) is an endocrine disruptor that is frequently adsorbed by MPs. Research indicates that under simulated biological gastrointestinal conditions, BPA can rapidly desorb from PS, PP, and PA MPs. This implies that when MPs are ingested by organisms, the BPA they carry can be quickly released, thereby exerting toxic effects on the organisms. The desorption rate may be influenced by gastric acid, digestive enzymes, and intestinal amphiphilic components such as bile salts, phospholipids and fatty acids. These components can behave as biological surfactants by forming micelles or modifying the MP surface, thereby changing the release and bioaccessibility of sorbed hydrophobic pollutants [79,80]. These factors can alter the surface properties of MPs, reduce the binding force between BPA and the MPs, and promote the release of BPA [79]. Ioannidis et al. investigated the desorption of uranium (U-232) and americium (Am-241) from polyamide nylon 6 (PN6) MPs under various conditions. The results demonstrated desorption of radionuclides from MPs in simulated human digestive fluids. The desorption mechanism, similar to that of the aforementioned pollutants, is also influenced by pH, ionic strength, and the internal environment of living organisms. This indicates that after microplastic ingestion, the radioactive substances they carry may be released within the body, increasing the risk of radiation exposure [81]. Eventually, MPs and the pollutants they carry can accumulate within organisms through desorption and be transmitted through the food chain, posing potential risks to the health of organisms and even humans [13,82,83,84].

#### 3.2.3. Pore-Filling Mechanism

MPs may contain surface defects, cracks, voids and pores generated during manufacture, mechanical abrasion, UV weathering or chemical oxidation. Pollutants can diffuse into these micro- or nanoscale free-volume regions and become retained when their molecular size and shape are compatible with the accessible voids. Therefore, pore filling should be interpreted broadly as retention within surface defects, pores or internal free-volume domains rather than as a universal regular pore network in all polymers [85]. For PS, the phenyl-substituted polymer chains may create hydrophobic free-volume regions and aromatic domains that favor retention of planar aromatic contaminants; however, the size and regularity of these domains depend strongly on polymer preparation, aging and physical damage, and should not be generalized as uniform pores formed solely by benzene-ring stacking [86,87].

### 3.3. Factors Influencing the Interaction Between MPs and Pollutants

**Figure 3:**
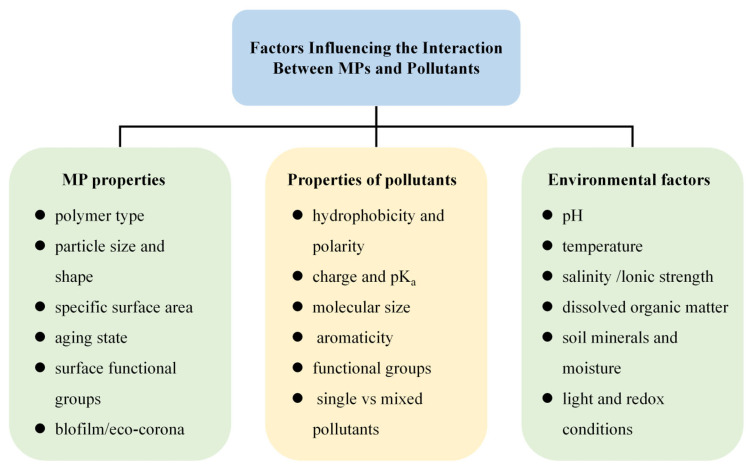
Main factors influencing interactions between MPs and pollutants, including MP properties, pollutant properties, and environmental factors.

The interaction between MPs and pollutants is a complex environmental issue, with the process influenced by a combination of factors including the structural properties of the MPs themselves, the nature of the pollutants, and environmental conditions (Figure 3).

#### 3.3.1. Structural Properties of MPs

The physicochemical properties of MPs, such as size, shape, density, surface charge, and surface functional groups, are key factors influencing their interactions with pollutants.

##### Types and Chemical Structures of Common MPs

Polymer type is a primary determinant of MP–pollutant interactions because it controls hydrophobicity, crystallinity, density, aromaticity, and the availability of functional groups or additives. The main polymer types discussed in environmental studies are summarized in Table 1.

##### Particle Size, Specific Surface Area, and Pore Structure

The particle size and specific surface area of MPs significantly influence their ability to adsorb pollutants. Generally, MPs with smaller particle sizes tend to possess larger specific surface areas. A larger specific surface area and a well-developed pore structure provide more adsorption sites, thereby enhancing the adsorption capacity of MPs for pollutants [85]. Studies on the adsorption behavior of TCS by PVC MPs have found that smaller PVC MPs (with larger specific surface areas) exhibit higher TCS adsorption capacity than larger PVC MPs [88]. This demonstrated that as microplastic particle size decreased, their specific surface area increased, thereby providing more adsorption sites and enhancing their ability to adsorb pollutants. However, excessively small particle size may also lead to particle agglomeration, which conversely reduces the effective adsorption area [89].

The pore structure of MPs is one of the key factors influencing their adsorption behavior toward pollutants. This structure primarily encompasses parameters such as pore size, pore volume, and specific surface area. Collectively, these characteristics determine the efficiency and mechanisms by which MPs function as pollutant carriers. It directly influences their ability to adsorb pollutant molecules into their interior. A more developed pore structure typically indicates a greater number of adsorption sites, facilitating easier diffusion and transport of pollutants within the MPs [90]. Especially for hydrophobic pollutants, the porous structure of MPs can provide additional hydrophobic adsorption sites, promoting their adsorption onto the microplastic surface. Additionally, pollutant molecules of different sizes may preferentially adsorb into specific pores matching their dimensions. For instance, larger pollutant molecules may more readily enter the macropores of MPs, while smaller molecules tend to accumulate in micropores and mesopores. Research on biomass-derived activated carbon adsorbents (BACAs) indicates that even with identical specific surface areas, differing pore size distributions significantly influence adsorption/desorption performance for volatile organic compounds (VOCs) such as benzene, toluene, and xylene [91]. For MPs, this pore-size matching effect is also widely observed, determining their selective adsorption capacity for different types of pollutants [92].

##### Polarity and Surface Functional Groups

The polarity and surface functional groups of MPs determine the types of interactions they form with pollutants, such as electrostatic forces, hydrogen bonding, hydrophobic interactions, and π–π interactions. The electrostatic attraction and repulsion are key manifestations of polar interactions. Generally speaking, MPs with stronger polarity tend to adsorb polar pollutants more readily, while those with higher hydrophobicity exhibit greater adsorption capacity for hydrophobic organic pollutants [93]. For example, the surfaces of MPs containing polar functional groups such as -OH, -COOH, or -NH_2_ groups can interact with polar pollutants through hydrogen bonding or electrostatic attraction. In contrast, for hydrophobic MPs (e.g., polyethylene, polypropylene), hydrophobic interactions serve as the primary mechanism for adsorbing organic pollutants [18,59,94]. Functional groups on the surface of MPs, particularly oxygen-containing functional groups generated during aging, play a crucial role in pollutant adsorption behavior. The functional groups formed on the surface of aged MPs, such as carboxyl and hydroxyl groups, can directly form surface complexes with heavy metal ions, thereby enhancing the adsorption of heavy metals. Research on aged PS and PVC MPs indicates that aging increases the presence of oxygen-containing functional groups on the microplastic surface, thereby enhancing their adsorption capacity for heavy metals [95]. Additionally, biofilms forming on microplastic surfaces can alter their surface properties by introducing new functional groups. Through bioaccumulation, these biofilms further influence pollutant adsorption. Functional groups within the biofilm, such as -COOH and -NH_2_, can form stable complexes with heavy metals, leading to their accumulation on the microplastic surface [14].

##### Degree of Aging

The degree of microplastic aging is crucial to its interactions with pollutants. In the environment, MPs undergo aging processes such as ultraviolet radiation, biodegradation, physical abrasion, and chemical oxidation. These processes alter the surface morphology, chemical structure, and hydrophilicity of MPs, consequently changing their adsorption capacity for pollutants (Table 2). With UV exposure, MPs become oxidized, forming additional oxygen-containing functional groups that alter their polarity and adsorption properties. Aged MPs may exhibit enhanced pollutant adsorption due to increased surface roughness and functional group changes [96]. Under ultraviolet irradiation, PS MPs develop a rough surface with pores and an increase in oxygen-containing functional groups, thereby altering their adsorption capacity for heavy metals [97]. After 30 days of UV aging, the surface oxygen content of PVC MPs increased, their hydrophobicity changed, and their adsorption of erythromycin (ERY) increased, while their adsorption of cypermethrin (CPF) decreased [98].

The effect of aging on microplastic adsorption of pollutants depends on the polymer type of the MPs, the aging method, the nature of the pollutants, and environmental conditions. For example, ultraviolet-aged PE and PS MPs exhibit enhanced adsorption capacity for tetracycline [103]. Chemical aging (Fenton oxidation) significantly enhanced the adsorption capacity of PE, PP, and PS MPs for triclosan [102]. And PE exhibits significantly enhanced adsorption capacity for Cu and Zn following H_2_O_2_ aging. In addition, research has also found that aged MPs might exhibit reduced or unchanged adsorption capacity for certain organic pollutants. This phenomenon may be attributed to the factors such as pollutant type, changes in microplastic surface polarity, and biofilm formation [104]. Finally, the aging process of MPs affects the composition of surface microbial communities, thereby altering the capacity to serve as carriers of antibiotic resistance genes (ARGs). Studies indicate that photoaging of MPs typically leads to higher abundances of ARGs on their surfaces, potentially due to aging altering the surface properties of MPs and biofilm formation [105].

##### Polymer Type and Morphology

Different types of MPs (such as polyethylene, polypropylene, polystyrene, polyvinyl chloride, polyethylene terephthalate, etc.) exhibit distinct physicochemical properties and adsorption capacities due to variations in their repeating-unit composition, degree of polymerization, crystallinity, and additives. For example, PS exhibit significant adsorption capacity for organic pollutants such as aniline due to their hydrophobic surface and aromatic ring structure [86]; MPs made from different materials such as low-density polyethylene (LDPE), polyethylene terephthalate (PET), and PVC also exhibit varying adsorption capacities for micropollutants in river environments [106]. Competitive adsorption studies of malachite green (MG) and rhodamine B (RhB) on PE and PVC MPs reveal competition among different pollutants for adsorption sites on MPs [107]. Additionally, since MPs of different shapes may exhibit varying surface roughness and exposed functional groups, their morphology, such as spherical, fibrous, or fragmentary forms, also influences their migration behavior in the environment and their efficiency in adsorbing pollutants [108].

#### 3.3.2. Properties of Pollutants

The pollutant side of the interaction is equally important. Hydrophobicity, polarity, molecular size, charge, aromaticity, and functional groups determine whether a compound is dominated by partitioning, surface complexation, electrostatic interactions, hydrogen bonding, π–π interactions, or pore filling.

##### Types of Pollutants

MPs interact with both organic and inorganic pollutants. Representative pollutant classes and their dominant interaction pathways are summarized in Table 3.

##### Polarity and Hydrophobicity

The surfaces and interstitial spaces of MPs contain polar functional groups and hydrophobic regions, and these characteristics determine their adsorption capacity for various chemical pollutants. On the one hand, polar pollutants readily interact with polar functional groups on the surface of MPs through hydrogen bonding or electrostatic forces. Research indicates that polar MPs such as polybutylene succinate (PBS) and polycaprolactone (PCL) typically exhibit stronger adsorption capacities than non-polar MPs (LDPE and PS). For a set of neutral organic compounds evaluated by Xu et al., the adsorption coefficients (Kd) for PBS and PCL ranged from 130 to 42,002 L/kg and 124 to 27,768 L/kg, respectively, whereas the Kd values for LDPE and PS ranged from 6.40 to 10,713 L/kg and 1.52 to 10,332 L/kg [109]. This indicates that polar MPs may form stronger interactions with polar pollutants through their surface polar groups. On the other hand, for hydrophobic pollutants, hydrophobic interactions serve as the primary mechanism for microplastic adsorption of contaminants, such as PAHs which tend to bind to the hydrophobic surface of MPs through hydrophobic forces [110], non-polar MPs such as PE and PP also exhibit strong adsorption capacity for hydrophobic organic pollutants [109]. The octanol-water partition coefficient (Kow) of a pollutant is a key indicator of its hydrophobicity. A higher Kow value indicates stronger hydrophobicity, greater adsorption capacity, and more pronounced hydrophobic interactions with MPs [111].

##### Charge and Functional Groups

The charge and functional groups of pollutants can influence their interactions with MPs through mechanisms such as electrostatic attraction, hydrogen bonding, and coordination. Microplastic surfaces typically carry charges, and their surface properties undergo changes, particularly after aging processes, introducing additional charged functional groups [101]. Electrostatic attraction occurs when pollutants and microplastic surfaces carry opposite charges. For example, cationic pollutants such as certain heavy metal ions or cationic pharmaceuticals tend to adsorb onto negatively charged microplastic surfaces. PS MPs with introduced carboxyl groups (PS-COOH), due to their negatively charged surfaces, can more effectively adsorb cationic pollutants, thereby reducing the combined cytotoxicity of these cationic contaminants [112]. Conversely, if pollutants and MPs carry the same surface charge, electrostatic repulsion occurs, thereby reducing adsorption efficiency. Additionally, pH alters the degree of ionization of surface functional groups on MPs, consequently affecting their surface charge [113]. Research indicates that in environments with higher pH levels (e.g., pH 7.0), microplastic surfaces may carry negative charges, while acidic organic pollutants may also undergo deprotonation to acquire negative charges. Under these conditions, electrostatic repulsion weakens adsorption forces [93].

Contaminant functional groups bind to MPs through various mechanisms, including hydrogen bonding, hydrophobic interactions, and π–π interactions, forming either specific or non-specific associations. Polar functional groups on contaminant molecules, such as hydroxyl, carboxyl, and amino groups, can form hydrogen bonds with polar groups present on microplastic surfaces, such as C-O and C=O bonds. For example, pharmaceutical molecules like SMZ contain groups capable of forming hydrogen bonds, which facilitates their adsorption onto MPs such as PP and PE [60]. MPs typically possess hydrophobic surfaces, allowing the hydrophobic components of pollutant molecules, such as aromatic rings and long-chain alkane structures, to bind to MPs through hydrophobic interactions. This mechanism explains the adsorption of PAHs and their derivatives onto MPs [114]. When both the pollutant molecules and the microplastic surfaces contain aromatic ring structures, π–π interactions can occur. This interaction typically takes place between conjugated systems. For example, certain drug molecules (such as carbamazepine) possess aromatic rings that can form π–π stacking interactions with the benzene ring structures of polystyrene MPs, thereby enhancing adsorption capacity [70]. The adsorption of benzene, phenanthrene, and their hydroxy derivatives onto PVC MPs also involves π–π interactions [93].

#### 3.3.3. Environmental Factors

Environmental conditions such as pH, temperature, salinity and organic matter content exert complex influences on the interactions between MPs and pollutants.

##### pH

Environmental pH alters MP–pollutant interactions by influencing the surface charge of MPs, the ionization state of organic pollutants, and the speciation of metal ions. In this review, acidic, near-neutral and alkaline conditions are operationally considered as pH < 5, pH 6–8 and pH > 8, respectively, although the exact boundary depends on the pKa of the pollutant and the point of zero charge (pHpzc) of the MP surface.

At low pH, H+ competes with metal cations for oxygen-containing binding sites and can suppress metal adsorption onto aged MPs. Near neutral pH, common polymers such as PE, PP and PS often carry negative surface charges and may adsorb cationic pharmaceuticals or metals more strongly. Under alkaline conditions, acidic organic pollutants such as sulfamethoxazole, chlorinated phenols and other phenolic compounds can deprotonate and become negatively charged, which may enhance electrostatic repulsion from negatively charged MPs; meanwhile, some metals may form hydroxide complexes or precipitates that change their apparent adsorption behavior.

##### Temperature and Salinity

Temperature is one of the key environmental factors influencing the adsorption behavior of pollutants on MPs. Firstly, elevated temperatures can affect both the adsorption capacity and adsorption rate of pollutants on MPs. For instance, studies have shown that in simulated digestive fluids, increased temperatures can alter the adsorption behavior of polychlorinated biphenyls (PCBs) from MPs [115]. Secondly, temperature alters the intermolecular forces between pollutant molecules and microplastic surfaces, such as hydrophobic interactions, hydrogen bonds, π–π interactions, and electrostatic forces. Elevated temperatures typically increase molecular motion, potentially promoting or inhibiting the adsorption process. Finally, elevated temperatures may alter the physicochemical properties of MPs themselves, thereby affecting their surface area and pore structure and consequently changing their adsorption capacity for pollutants. For example, the adsorption behavior of polystyrene MPs toward aniline is influenced by temperature [86].

Salinity is another critical environmental factor, with variations significantly influencing microplastic behavior and their interactions with pollutants. Increased salinity elevates ionic strength, compresses the electrical double layer, promotes aggregation of some MPs, and reduces the solubility of hydrophobic organic compounds through a salting-out effect. These changes can facilitate the transfer of hydrophobic pollutants from water to MP surfaces [116]. Salt ions, especially divalent cations, may also compete with pollutants for adsorption sites or form cation bridges between negatively charged MPs and anionic pollutants. For example, high salinity alters the adsorption behavior of PS and PA MPs toward lead, cadmium, and tetracycline [117]. In coastal wetlands and saline soils, the statement that salinity limits vertical migration means that salt-induced aggregation, changes in pore-water chemistry, and enhanced retention within soil pores may reduce downward transport of MPs, causing stronger accumulation in surface layers and increasing local contamination risks [118].

##### Organic Matter and Humus

Organic matter and humic substances in the environment significantly influence the interactions between MPs and various pollutants, such as organic contaminants and heavy metals. The presence of organic matter and humic substances plays a complex dual role in microplastic-pollutant interactions, potentially promoting or inhibiting pollutant adsorption and desorption. Organic matter can alter the surface energy and zeta potential of MPs, thereby affecting their colloidal stability and consequently influencing their sedimentation, aggregation, and transport in aquatic environments. Zhang et al. found that the presence of organic matter significantly reduced the interparticle forces between polystyrene MPs, thereby affecting their aggregation and deposition within marine ecosystems [119]. The meta-analysis by An et al. should be interpreted as evidence that the effect of MPs on metal bioavailability in soil is context-dependent: the magnitude of MP-induced changes increased with MP size, soil sand content, and exposure duration, but decreased as soil pH and soil organic matter increased. In other words, organic matter may buffer the influence of MPs on metal bioavailability by providing additional binding phases and reducing the relative contribution of plastic surfaces [120].

Humus, as the primary component of natural organic matter, possesses a molecular structure rich in polar functional groups such as carboxyl and phenolic hydroxyl groups. These groups can influence the adsorption of pollutants by MPs through multiple mechanisms [121]. On one hand, humic substances can form an “eco-corona” or bioaggregates on the surface of MPs, altering their surface properties such as surface charge, hydrophobicity, and functional group types. On the other hand, humic substances may also inhibit the adsorption of pollutants onto MPs by competing for adsorption sites or forming soluble complexes [122]. For example, Chen et al. found that in the absence of MPs, PCBs and their hydroxylated derivatives (hydroxy PCBs) exhibited strong affinity for humic acid. However, in the presence of MPs, the binding capacity of PCBs and hydroxy PCBs to humic acid was significantly reduced, suggesting that MPs may influence the binding between organic pollutants and natural organic matter [123]. This competitive adsorption mechanism allows humus to potentially reduce the efficiency of MPs as pollutant carriers.

### 3.4. Biological and Ecological Effects of MP–Pollutant Interactions

MPs not only possess inherent ecological toxicity but also act as carriers that adsorb organic pollutants in the environment, forming a composite pollution system. This significantly alters the environmental behavior of pollutants and generates ecological toxicity effects [124].

#### 3.4.1. Impacts of MPs on the Environmental Behavior of Organic Pollutants

##### Migration Behavior Changes

MPs’ strong adsorption properties and high mobility in the environment make them carriers for the migration and dissemination of pollutants. Research has demonstrated that MPs not only exhibit a high capacity for accumulating organic pollutants but also significantly influence the environmental migration of pollutants, as well as their accumulation and transfer within organisms [125].

In aquatic environments, MPs alter the migration capacity of organic pollutants through physical encapsulation and surface adsorption. Studies indicate a significant correlation between the abundance of MPs in organisms and their impact on pollutant migration. Smaller MPs more readily adsorb organic pollutants, leading to increased contaminant accumulation upon biological ingestion. MPs measuring 30–500 μm exhibit higher affinity for adsorbing 4–5-ring PAHs, while those between 0.5–2 mm show stronger migration correlations with 2-ring PAHs. However, MPs larger than 2 mm demonstrate no correlation with PAHs [126]. Additionally, the density of MPs also influences the vertical migration of pollutants. For instance, high-density MPs like polytetrafluoroethylene (PTFE) exhibit higher accumulation levels in deep-sea ecosystems [127].

In soil environments, MPs alter the physicochemical properties of soil by affecting its pore structure and water retention capacity. This indirectly regulates the migration pathways of organic pollutants and also influences their migration behavior within the soil. Research has found that the hydrophobic surface of MPs can adsorb more organic pollutants, promoting both horizontal and vertical migration of these contaminants through their movement within the soil [128]. The presence of MPs can also enhance the mobility of certain antibiotics in soil. For instance, in sandy soils, polyamide (PA) MPs significantly increased the migration capacity of oxytetracycline (OTC) [129]. Additionally, the interaction between MPs and soil minerals can also influence the migration processes of organic pollutants. For example, clay minerals (montmorillonite and kaolinite) can provide adsorption sites for thiamethoxam while also occupying adsorption sites on MPs through van der Waals forces, hydrogen bonding, and polar interactions, thereby altering thiamethoxam’s migration capacity in soil [130].

##### Transformation and Degradation Processes

The interaction between MPs and organic pollutants also involves transformation and degradation processes. Under specific conditions, such as ultraviolet radiation, MPs may promote the transformation of pollutants. Research has found that microplastic-derived dissolved organic matter (PDOM) can selectively degrade organic pollutants in aquatic environments through a molecular photoresponse sequence, generating reactive oxygen species (ROS) to remove organic contaminants [131]. Additionally, the adsorption of organic pollutants by MPs may influence natural degradation processes such as photolysis or hydrolysis. For instance, in suspended particulate-water systems, the presence of polypropylene (PP) MPs significantly alters the photochemical transformation behavior of octachlorodibenzo-p-dioxin (OCDF). PP enhances the photodegradation rate of OCDF by increasing reactive oxygen species (ROS) concentrations [132].

##### Bioaccumulation Effects

MPs significantly influence the bioaccumulation of organic pollutants in the environment. When organisms ingest MPs coated with organic pollutants, the pollutants may desorb from the MPs and accumulate within the organisms, leading to higher pollutant concentrations in biological tissues compared to microplastic-free control groups [133]. Research indicates that MPs may enhance the bioaccumulation of organic pollutants in aquatic organisms [134]. However, some studies suggest that MPs may reduce the bioaccumulation of organic pollutants in fish by adsorbing these pollutants and decreasing their bioavailability. This variation may be related to the characteristics of the MPs, the types of organic pollutants, and the physiological traits of the organisms [135]. Furthermore, MPs and the organic pollutants adsorbed onto them can be transferred through the food chain and bioaccumulated from lower trophic levels to higher ones, ultimately posing a threat to human health [13,124,136,137].

#### 3.4.2. Toxic Effects on Different Biological Groups

##### Aquatic Organisms

Due to the widespread presence of MPs in aquatic environments, aquatic organisms serve as the primary receptors for the combined toxicity of MPs and organic pollutants. Research indicates that the interaction between MPs and organic pollutants may produce either synergistic or antagonistic effects on the toxicity to aquatic organisms. For instance, prolonged exposure to the PE and glyphosate (GLY) system reduces the swimming speed of carp and causes significant alterations in intestinal metabolism, microbial abundance, and diversity, while also disrupting the physical barrier [138]. In another study, zebrafish fed low-density polyethylene (LDPE) MPs adsorbing organic pollutants (polychlorinated biphenyls, brominated flame retardants, perfluorinated compounds, and methylmercury) exhibited significantly disrupted homeostasis in their liver, brain, gut, and muscle tissues. Granular white patches and elevated concentrations of organic pollutants were also observed in zebrafish livers, indicating that the interaction between MPs and organic pollutants enhances biological toxicity [139]. However, MPs may reduce the concentration of organic pollutants in water through partitioning or adsorption, thereby decreasing the toxicity of organic pollutants. Zhang et al. investigated the combined effects of PS and roxithromycin (ROX) on freshwater Nile tilapia, indicating that co-exposure to PS and ROX significantly reduced liver malondialdehyde (MDA) levels and increased superoxide dismutase (SOD) activity. This suggests that the presence of MPs may mitigate oxidative damage to the liver and potentially reduce the neurotoxicity of ROX on the cholinergic system [140].

##### Soil Organisms

Soil environments also serve as significant reservoirs for MPs, and combined exposure to organic pollutants in soil can impact soil organisms. Beyond carrier effects, MPs themselves can affect earthworms, nematodes, collembolans, mites, plants and soil microorganisms. Reported responses include gut abrasion, reduced growth and reproduction, oxidative stress, altered antioxidant enzyme activity, changes in microbial community structure, disruption of nutrient cycling, and changes in soil aggregation and water transport [141,142].

When MPs co-occur with pesticides, heavy metals or antibiotics, their toxicological effects become more complex. MPs may increase pollutant exposure by transporting sorbed compounds into the gut of soil organisms, but they may also reduce freely dissolved concentrations by sequestration. This dual behavior explains why synergistic, additive and antagonistic toxicity have all been reported. For soil ecological risk assessment, it is therefore important to evaluate MP polymer type, size, aging state, pollutant mixture composition and biofilm development rather than MP abundance alone [143].

##### Mammals and Human Health

The risks posed by MPs to mammalian and human health primarily stem from ingestion through food and drinking water, inhalation of airborne particles, and, to a lesser extent, dermal or occupational exposure. Major contamination sources include bottled and tap water, seafood, salt, food packaging, indoor dust, textile fibers, tire and road-wear particles, and atmospheric deposition [137,143,144]. MPs have been detected in human-related samples such as feces, blood, placenta, sputum, saliva and lung tissue, suggesting that exposure is widespread, although dose–response relationships and long-term disease causality remain uncertain [143,144].

In vitro studies using intestinal, lung, immune, hepatic and neuronal cell models indicate that MPs and MNPs can induce cellular uptake, oxidative stress, inflammatory responses, mitochondrial dysfunction, membrane damage, altered gene expression, apoptosis or autophagy, depending on particle size, polymer type, surface charge, concentration and exposure duration [143,145]. These findings support a biologically plausible basis for human health concern, but extrapolation from cell models to realistic human exposure requires caution.

Nanoplastics may pose greater potential hazards than larger MPs because their smaller size, larger specific surface area and higher mobility make them more likely to penetrate physiological barriers. Experimental studies suggest that small MNPs can cross epithelial barriers and may reach systemic circulation; evidence from animal and cell models also indicates potential interactions with the blood–brain barrier. Therefore, this review clarifies that BBB penetration is more plausible for nanoscale plastics than for larger MPs, while direct evidence for routine penetration of the human BBB by environmental MPs remains limited [143,146].

## 4. Implications for Sustainable Environmental Management and Mitigation

From the perspective of sustainable environmental systems, the significance of MP–pollutant interactions extends beyond mechanistic complexity, because these interactions directly affect pollution prevention, wastewater treatment, sludge reuse, agricultural soil quality, and long-term ecological risk management. MPs are not isolated contaminants, but mobile carriers that can alter the environmental fate, bioavailability, and toxicity of co-existing pollutants through adsorption, desorption, and trophic transfer [21,147]. At the source-control stage, reducing releases of both primary and secondary MPs remains the most effective long-term strategy.

Wastewater-related emissions, agricultural plastics, tire wear, landfill leachate, and diffuse urban sources have all been identified as important pathways introducing MPs into environmental systems [17,38,148]. Wastewater treatment plants should be considered key control nodes within sustainable environmental systems. Although treatment processes can retain a substantial proportion of MPs, they may also transfer them from the water phase to sewage sludge rather than eliminating the overall burden [38,149]. This implies that sludge recycling and land application should be evaluated not only from a nutrient recovery perspective, but also in terms of their role in redistributing MPs and associated contaminants to agricultural soils [33,149].

In soil systems, management should move beyond simple abundance-based assessment. MPs can interact with natural organic matter, influence contaminant sorption behavior, and modify pollutant mobility and ecological exposure under different environmental conditions [122].

For aquatic and estuarine environments, mitigation strategies should account for the dynamic nature of MP–pollutant interactions. Existing studies show that environmental variables such as salinity, temperature, and dissolved organic matter may substantially alter adsorption behavior, desorption potential, and contaminant transport [116]. This means that the ecological risks associated with MPs are not static, but may evolve across environmental gradients and with weathering or aging processes. Accordingly, environmental monitoring programs should move beyond simple MP quantification and include polymer type, aging status, associated pollutants, and relevant hydro chemical parameters [104]. Such a monitoring framework would provide a more realistic basis for risk assessment and management in complex environments such as estuaries, coastal wetlands, and mixed land–sea interfaces [118].

### Environmental Education and Stakeholder Participation

Environmental education is also an essential component of mitigation. Public understanding of MP sources can reduce unnecessary single-use plastic consumption, improve waste sorting and recycling behavior, encourage proper disposal of fishing gear and agricultural films, and increase awareness of textile-fiber release, tire-wear particles, and indoor dust exposure. Education programs can also support citizen-science monitoring and help translate scientific findings into practical behavior changes at household, school, industrial and municipal levels.

For managers, environmental education should be integrated with source-control policies, product design, wastewater and sludge management, and monitoring programs. Such integration is particularly important because MPs are not only a waste-management issue but also a cross-media pollutant carrier that links human behavior, material use, ecosystem exposure and long-term health risk.

Overall, sustainable management of MP–pollutant interactions requires a shift from pollutant-specific control toward integrated system governance that connects source reduction, wastewater and sludge management, agricultural practice optimization, and long-term monitoring [21]. Such a framework would better support environmental protection under broader goals of circularity, resource efficiency, and sustainable land and water management.

## 5. Conclusions and Outlooks

MPs are widespread contaminants that act not only as particles with their own ecological risks, but also as mobile surfaces that can carry, release, and transform other pollutants. The main value of this review is to connect these scattered processes into a single source–mechanism–effect–management chain. Within this chain, water, soil, and air are not treated as isolated compartments, because MPs and their associated pollutants can move among them through wastewater discharge, sludge reuse, agricultural plastic residues, landfill leachate, tire wear, textile fibers, runoff, and atmospheric deposition. This perspective helps explain why the risk of MPs cannot be judged only by particle abundance, but should also consider polymer type, aging status, pollutant mixtures, biofilm development, desorption potential, biological uptake, and the environmental conditions that control these processes.

The interaction between MPs and pollutants is controlled by partitioning, surface adsorption, desorption, pore filling, and biofilm-related processes. These mechanisms are not universal; their relative importance changes with polymer type, particle size, crystallinity, aging state, pollutant hydrophobicity, charge, pKa, molecular size, pH, salinity, temperature, and organic matter. The revised review therefore emphasizes matrix-specific regulation, including salinity and DOM effects in aquatic systems, organic matter and pore-structure effects in soils, and atmospheric aging and deposition in air.

MP–pollutant interactions can modify pollutant migration, degradation, bioaccumulation, trophic transfer, and toxicity. Depending on environmental conditions, MPs may either enhance exposure by acting as carriers and releasing pollutants in organisms, or reduce freely dissolved pollutant concentrations through adsorption. This dual role explains why risk assessment should not rely only on MP abundance, but should also consider polymer identity, aging status, associated pollutant load, environmental matrix, and exposure route.

Future work should prioritize environmentally realistic multi-factor experiments, standardized methods for airborne and soil MPs, quantitative desorption data under digestive and rhizosphere conditions, and stronger integration of MP and NP toxicology. In particular, future studies should test how pollutant mixtures, biofilms, aging history and environmental gradients jointly determine whether MPs act mainly as pollutant vectors, pollutant sinks, or reactive interfaces. From a management perspective, the most urgent outlook is to reduce unnecessary plastic use, improve plastic product design and end-of-life collection, prevent releases from wastewater, sludge, mulch films, textiles and tire wear, and mitigate the ecological effects already detected through targeted remediation, public education and long-term risk-informed monitoring.

## Figures and Tables

**Table 1:** Common MP polymer types, representative repeating units, and properties relevant to pollutant interactions.

Polymer	Abbreviation	Representative Repeating Unit/Structural Feature	Main Interaction-Relevant Features
Polyethylene	PE	[-CH_2_-CH_2_-]n	Nonpolar, highly hydrophobic, low density; strong partitioning of hydrophobic organic pollutants.
Polypropylene	PP	[-CH_2_-CH(CH_3_)-]n	Nonpolar and hydrophobic; high affinity for hydrophobic compounds, but aging-induced oxidation changes behavior.
Polystyrene	PS	[-CH_2_-CH(C_6_H_5_)-]n	Aromatic phenyl groups support π–π interactions with aromatic pollutants.
Polyvinyl chloride	PVC	[-CH_2_-CHCl-]n	Polar C-Cl bonds and additives; adsorption influenced by polarity, aging and plasticizers.
Polyethylene terephthalate	PET	[-O-CH_2_-CH_2_-O-CO-C_6_H_4_-CO-]n	Ester groups and aromatic rings; hydrogen bonding and π–π interactions may occur.
Polyamide/nylon	PA	e.g., [-NH-(CH_2_)_5_-CO-]n	Amide groups favor hydrogen bonding and interactions with polar pollutants or metals.
Polyurethane	PU	[-NH-CO-O-R-]n	Urethane groups and variable soft/hard segments; surface polarity depends on formulation.

Note: The repeating units are simplified text representations of idealized polymer structures; commercial plastics may also contain additives, fillers, dyes, stabilizers, and weathering products that affect pollutant interactions.

**Table 2:** Effects of aging methods on MP–pollutant interactions.

Aging Method	MP Type	Pollutant(s)	Influence on Interaction Between MPs and Pollutants	Reference(s)
UV aging	PS	BDE-47	Aging reduced BDE-47 adsorption relative to pristine PS, indicating that oxidation may weaken hydrophobic sorption for some brominated flame retardants.	[99]
UV aging	PS	Erythromycin (ERY)	UV-aged PS showed increased ERY adsorption and altered biofilm formation and antibiotic-resistance mutation.	[100]
UV aging	PVC	Chlorpyrifos (CPF), ERY	Aging increased ERY adsorption but decreased CPF adsorption, demonstrating pollutant-specific responses.	[98]
High-temperature aging	PS	Sulfamethazine (SMT), naphthalene (NAP), phenanthrene (PHE)	Aging increased adsorption capacity by modifying surface roughness, polarity and polymer structure.	[101]
Electron-beam aging	PP	NAP, Pb^2+^	Oxygen-containing groups enhanced metal binding, whereas organic adsorption depended on surface polarity and pollutant hydrophobicity.	[87]
Chemical oxidation	PE, PP, PS	Triclosan and selected metals	Oxidative aging generally introduced oxygen-containing functional groups and strengthened specific surface interactions.	[102]

Abbreviations: PS, polystyrene; PVC, polyvinyl chloride; PP, polypropylene; PE, polyethylene; BDE-47, 2,2′,4,4′-tetrabromodiphenyl ether; ERY, erythromycin; CPF, chlorpyrifos; SMT, sulfamethazine; NAP, naphthalene; PHE, phenanthrene.

**Table 3:** Major pollutant classes interacting with MPs and typical interaction mechanisms.

Pollutant Class	Representative Examples	Typical Interaction Mechanisms with MPs
Persistent organic pollutants	PAHs, PCBs, PBDEs, DDT	Hydrophobic partitioning, π–π interactions and pore filling.
Pharmaceuticals and antibiotics	Tetracycline, ciprofloxacin, sulfamethoxazole, carbamazepine	pH-dependent electrostatic interactions, hydrogen bonding, cation bridging and π–π interactions.
Pesticides and herbicides	Glyphosate, chlorpyrifos, atrazine	Hydrophobic interactions, hydrogen bonding and mineral/organic-matter-mediated co-sorption.
Heavy metals and metalloids	Pb^2+^, Cd^2+^, Cu^2+^, Zn^2+^, As species	Complexation with oxygen-containing groups, electrostatic interactions and biofilm/EPS binding.
Endocrine-disrupting chemicals	BPA, phthalates, triclosan	Hydrophobic partitioning, hydrogen bonding and additive-related release from plastics.
Atmospheric co-contaminants	PAHs, tire-wear additives, soot-associated organics	Co-deposition, surface adsorption, aging-mediated oxidation and photochemical reactions.

## Data Availability

No new data were created or analyzed in this study. Data sharing is not applicable to this review article.

## References

[1] Lamoree M.H., van Boxel J., Nardella F., Houthuijs K.J., Brandsma S.H., Béen F., van Duursen M.B.M. (2025). Health impacts of microplastic and nanoplastic exposure. Nat. Med..

[2] Lin Z.R., Hu X.Y., Lin H.J., Yu G.Y., Shen L.G., Yu W., Li B.S., Zhao L.H., Ying M.Y. (2025). Membrane technology for microplastic removal: Microplastic occurrence, challenges, and innovations of process and materials. Chem. Eng. J..

[3] Qin L., Qian S.X., Zhou X.R., Al-Dhabi N.A., Lai C., Ma D.S., Huo X.Q., Wu L., Tang W.W. (2024). Co-pyrolyzed biochar derived from microplastics and microalgae as peroxymonosulfate activator: Influence of microplastic types and analysis of systemic causes. J. Environ. Chem. Eng..

[4] Zhang K., Hamidian A.H., Tubic A., Zhang Y., Fang J.K.H., Wu C.X., Lam P.K.S. (2021). Understanding plastic degradation and microplastic formation in the environment: A review. Environ. Pollut..

[5] Rochman C.M., Hoellein T. (2020). The global odyssey of plastic pollution. Science.

[6] MacLeo M., Arp H.P.H., Tekman M.B., Jahnke A. (2021). The global threat from plastic pollution. Science.

[7] Li J., Liu H., Chen J.P. (2018). Microplastics in freshwater systems: A review on occurrence, environmental effects, and methods for microplastics detection. Water Res..

[8] Cole M., Lindeque P., Halsband C., Galloway T.S. (2011). Microplastics as contaminants in the marine environment: A review. Mar. Pollut. Bull..

[9] Thompson R.C., Moore C.J., vom Saal F.S., Swan S.H. (2009). Plastics, the environment and human health: Current consensus and future trends. Philos. Trans. R. Soc. B-Biol. Sci..

[10] Eerkes-Medrano D., Thompson R.C., Aldridge D.C. (2015). Microplastics in freshwater systems: A review of the emerging threats, identification of knowledge gaps and prioritisation of research needs. Water Res..

[11] Vethaak A.D., Legler J. (2021). Microplastics and human health. Science.

[12] Xiao S.L., Cui Y.F., Brahney J., Mahowald N.M., Li Q. (2023). Long-distance atmospheric transport of microplastic fibres influenced by their shapes. Nat. Geosci..

[13] Rafa N., Ahmed B., Zohora F., Bakya J., Ahmed S., Ahmed S.F., Mofijur M., Chowdhury A.A., Almomani F. (2024). Microplastics as carriers of toxic pollutants: Source, transport, and toxicological effects. Environ. Pollut..

[14] Liu B., Zhao S., Qiu T., Cui Q., Yang Y., Li L., Chen J., Huang M., Zhan A., Fang L. (2024). Interaction of microplastics with heavy metals in soil: Mechanisms, influencing factors and biological effects. Sci. Total Environ..

[15] Li J., Zhang K., Zhang H. (2018). Adsorption of antibiotics on microplastics. Environ. Pollut..

[16] Wang F., Zhang M., Sha W., Wang Y., Hao H., Dou Y., Li Y. (2020). Sorption Behavior and Mechanisms of Organic Contaminants to Nano and Microplastics. Molecules.

[17] Huang Y., Liu Q., Jia W., Yan C., Wang J. (2020). Agricultural plastic mulching as a source of microplastics in the terrestrial environment. Environ. Pollut..

[18] Fu L.N., Li J., Wang G.Y., Luan Y.N., Dai W. (2021). Adsorption behavior of organic pollutants on microplastics. Ecotoxicol. Environ. Saf..

[19] Luo H.W., Liu C.Y., He D.Q., Xu J., Sun J.Q., Li J., Pan X.L. (2022). Environmental behaviors of microplastics in aquatic systems: A systematic review on degradation, adsorption, toxicity and biofilm under aging conditions. J. Hazard. Mater..

[20] Hu L.H., Feng X.Y., Lan Y.Z., Zhang J.F., Nie P.H., Xu H.Y. (2024). Co-exposure with cadmium elevates the toxicity of microplastics: Trojan horse effect from the perspective of intestinal barrier. J. Hazard. Mater..

[21] Wang C.H., Zhao J., Xing B.S. (2021). Environmental source, fate, and toxicity of microplastics. J. Hazard. Mater..

[22] Menéndez-Pedriza A., Jaumot J. (2020). Interaction of Environmental Pollutants with Microplastics: A Critical Review of Sorption Factors, Bioaccumulation and Ecotoxicological Effects. Toxics.

[23] Xiang Y., Jiang L., Zhou Y., Luo Z., Zhi D., Yang J., Lam S.S. (2022). Microplastics and environmental pollutants: Key interaction and toxicology in aquatic and soil environments. J. Hazard. Mater..

[24] Mousazadehgavan M. (2026). Microplastics in Aquatic Systems: Dual Roles as Pollutant Carriers and Emerging Functional Materials for Water Treatment. Water Air Soil Pollut..

[25] de Carvalho J.G.R., Augusto H.C., Ferraz R., Delerue-Matos C., Fernandes V.C. (2024). Micro(nano)plastic and Related Chemicals: Emerging Contaminants in Environment, Food and Health Impacts. Toxics.

[26] Thompson R.C., Olsen Y., Mitchell R.P., Davis A., Rowland S.J., John A.W.G., McGonigle D., Russell A.E. (2004). Lost at sea: Where is all the plastic?. Science.

[27] Osman A.I., Hosny M., Eltaweil A.S., Omar S., Elgarahy A.M., Farghali M., Yap P.-S., Wu Y.-S., Nagandran S., Batumalaie K. (2023). Microplastic sources, formation, toxicity and remediation: A review. Environ. Chem. Lett..

[28] Auta H.S., Emenike C.U., Fauziah S.H. (2017). Distribution and importance of microplastics in the marine environment: A review of the sources, fate, effects, and potential solutions. Environ. Int..

[29] Briain O.O., Mendes A.R.M., McCarron S., Healy M.G., Morrison L. (2020). The role of wet wipes and sanitary towels as a source of white microplastic fibres in the marine environment. Water Res..

[30] De Witte B., Devriese L., Bekaert K., Hoffman S., Vandermeersch G., Cooreman K., Robbens J. (2014). Quality assessment of the blue mussel (Mytilus edulis): Comparison between commercial and wild types. Mar. Pollut. Bull..

[31] Cole M., Lindeque P.K., Fileman E., Clark J., Lewis C., Halsband C., Galloway T.S. (2016). Microplastics Alter the Properties and Sinking Rates of Zooplankton Faecal Pellets. Environ. Sci. Technol..

[32] Carr S.A., Liu J., Tesoro A.G. (2016). Transport and fate of microplastic particles in wastewater treatment plants. Water Res..

[33] Nizzetto L., Futter M., Langaas S. (2016). Are Agricultural Soils Dumps for Microplastics of Urban Origin?. Environ. Sci. Technol..

[34] Corradini F., Meza P., Eguiluz R., Casado F., Huerta-Lwanga E., Geissen V. (2019). Evidence of microplastic accumulation in agricultural soils from sewage sludge disposal. Sci. Total Environ..

[35] Zhang Y., Kang S., Allen S., Allen D., Gao T., Sillanpää M. (2020). Atmospheric microplastics: A review on current status and perspectives. Earth-Sci. Rev..

[36] Dris R., Gasperi J., Mirande C., Mandin C., Guerrouache M., Langlois V., Tassin B. (2017). A first overview of textile fibers, including microplastics, in indoor and outdoor environments. Environ. Pollut..

[37] Kole P.J., Lohr A.J., Van Belleghem F.G.A.J., Ragas A.M.J. (2017). Wear and Tear of Tyres: A Stealthy Source of Microplastics in the Environment. Int. J. Environ. Res. Public Health.

[38] Prata J.C. (2018). Microplastics in wastewater: State of the knowledge on sources, fate and solutions. Mar. Pollut. Bull..

[39] Abbasi S., Keshavarzi B., Moore F., Turner A., Kelly F.J., Dominguez A.O., Jaafarzadeh N. (2019). Distribution and potential health impacts of microplastics and microrubbers in air and street dusts from Asaluyeh County, Iran. Environ. Pollut..

[40] Liu F.F., Liu G.Z., Zhu Z.L., Wang S.C., Zhao F.F. (2019). Interactions between microplastics and phthalate esters as affected by microplastics characteristics and solution chemistry. Chemosphere.

[41] Wang F., Shih K.M., Li X.Y. (2015). The partition behavior of perfluorooctanesulfonate (PFOS) and perfluorooctanesulfonamide (FOSA) on microplastics. Chemosphere.

[42] Zhang H.B., Wang J.Q., Zhou B.Y., Zhou Y., Dai Z.F., Zhou Q., Christie P., Luo Y.M. (2018). Enhanced adsorption of oxytetracycline to weathered microplastic polystyrene: Kinetics, isotherms and influencing factors. Environ. Pollut..

[43] Xu B., Liu F., Brookes P.C., Xu J. (2018). The sorption kinetics and isotherms of sulfamethoxazole with polyethylene microplastics. Mar. Pollut. Bull..

[44] Chen B.L., Johnson E.J., Chefetz B., Zhu L.Z., Xing B.S. (2005). Sorption of polar and nonpolar aromatic organic contaminants by plant cuticular materials: Role of polarity and accessibility. Environ. Sci. Technol..

[45] Zhang P., Huang P., Sun H.W., Ma J.L., Li B.X. (2020). The structure of agricultural microplastics (PT, PU and UF) and their sorption capacities for PAHs and PHE derivates under various salinity and oxidation treatments. Environ. Pollut..

[46] Teuten E.L., Saquing J.M., Knappe D.R.U., Barlaz M.A., Jonsson S., Bjorn A., Rowland S.J., Thompson R.C., Galloway T.S., Yamashita R. (2009). Transport and release of chemicals from plastics to the environment and to wildlife. Philos. Trans. R. Soc. B-Biol. Sci..

[47] Amelia T.S.M., Khalik W.M.A.W.M., Ong M.C., Shao Y.T., Pan H.-J., Bhubalan K. (2021). Marine microplastics as vectors of major ocean pollutants and its hazards to the marine ecosystem and humans. Prog. Earth Planet. Sci..

[48] Bakir A., Rowland S.J., Thompson R.C. (2012). Competitive sorption of persistent organic pollutants onto microplastics in the marine environment. Mar. Pollut. Bull..

[49] Rai P.K., Sonne C., Brown R.J.C., Younis S.A., Kim K.-H. (2022). Adsorption of environmental contaminants on micro- and nano-scale plastic polymers and the influence of weathering processes on their adsorptive attributes. J. Hazard. Mater..

[50] Dabrowski A. (2001). Adsorption—From theory to practice. Adv. Colloid Interface Sci..

[51] Wang J., Tan Z., Peng J., Qiu Q., Li M. (2016). The behaviors of microplastics in the marine environment. Mar. Environ. Res..

[52] Guo X., Pang J., Chen S., Jia H. (2018). Sorption properties of tylosin on four different microplastics. Chemosphere.

[53] Wu C., Zhang K., Huang X., Liu J. (2016). Sorption of pharmaceuticals and personal care products to polyethylene debris. Environ. Sci. Pollut. Res..

[54] Xu B., Liu F., Brookes P.C., Xu J. (2018). Microplastics play a minor role in tetracycline sorption in the presence of dissolved organic matter. Environ. Pollut..

[55] Zhou Z., Sun Y., Wang Y., Yu F., Ma J. (2022). Adsorption behavior of Cu(II) and Cr(VI) on aged microplastics in antibiotics-heavy metals coexisting system. Chemosphere.

[56] Razanajatovo R.M., Ding J., Zhang S., Jiang H., Zou H. (2018). Sorption and desorption of selected pharmaceuticals by polyethylene microplastics. Mar. Pollut. Bull..

[57] Ahmed U., Sundholm D., Johansson M.P. (2024). The effect of hydrogen bonding on the π depletion and the π-π stacking interaction. Phys. Chem. Chem. Phys..

[58] Wang K., Kou Y., Guo C., Wang K., Li J., Schmidt J., Wang M., Liang S., Wang W., Lu Y. (2024). Comparison of rhodamine B adsorption and desorption on the aged non-degradable and degradable microplastics: Effects of charge-assisted hydrogen bond and underline mechanism. Environ. Technol. Innov..

[59] Upadhyay R., Singh S., Kaur G. (2022). Sorption of pharmaceuticals over microplastics’ surfaces: Interaction mechanisms and governing factors. Environ. Monit. Assess..

[60] Kuang B., Chen X., Zhan J., Zhou L., Zhong D., Wang T. (2023). Interaction behaviors of sulfamethoxazole and microplastics in marine condition: Focusing on the synergistic effects of salinity and temperature. Ecotoxicol. Environ. Saf..

[61] Guo C., Wang L., Lang D., Qian Q., Wang W., Wu R., Wang J. (2023). UV and chemical aging alter the adsorption behavior of microplastics for tetracycline. Environ. Pollut..

[62] Wang K., Kou Y., Wang K., Guo C., Liang X., Wang M., Li J., Liang S., Wang W., Wang J. (2025). Comparison of adsorption of seven ionic organic pollutants on polystyrene and poly(butylene adipate-co-terephthalate) microplastics: UV aging mechanism and role of charge-assisted hydrogen bond. Sep. Purif. Technol..

[63] Prajapati A., Vaidya A.N., Kumar A.R. (2022). Microplastic properties and their interaction with hydrophobic organic contaminants: A review. Environ. Sci. Pollut. Res..

[64] Cortes-Arriagada D., Miranda-Rojas S., Camarada M.B., Ortega D.E., Alarcon-Palacio V.B. (2023). The interaction mechanism of polystyrene microplastics with pharmaceuticals and personal care products. Sci. Total Environ..

[65] Cortes-Arriagada D., Ortega D.E. (2023). Interaction mechanism of triclosan on pristine microplastics. Sci. Total. Environ..

[66] Yang L., Hu J., Bai K. (2016). Capillary and van der Waals force between microparticles with different sizes in humid air. J. Adhes. Sci. Technol..

[67] Pakarinen O.H., Mativetsky J.M., Gulans A., Puska M.J., Foster A.S., Grutter P. (2009). Role of van der Waals forces in the adsorption and diffusion of organic molecules on an insulating surface. Phys. Rev. B.

[68] Kelkkanen A.K., Lundqvist B.I., Norskov J.K. (2011). Van der Waals effect in weak adsorption affecting trends in adsorption, reactivity, and the view of substrate nobility. Phys. Rev. B.

[69] Zhang J., Chen H., He H., Cheng X., Ma T., Hu J., Yang S., Li S., Zhang L. (2020). Adsorption behavior and mechanism of 9-Nitroanthracene on typical microplastics in aqueous solutions. Chemosphere.

[70] Wang Z., Wang Y., Yu K., Zhang M., Ding T., Xu L. (2024). Insights into the adsorption behavior of tetracycline in various shaped carbon nanopores: Interplay between mass transfer and adsorption. Microporous Mesoporous Mater..

[71] Wang K., Guo C., Li J., Wang K., Liang S., Wang W., Wang J. (2024). A critical review of the adsorption-desorption characteristics of antibiotics on microplastics and their combined toxic effects. Environ. Technol. Innov..

[72] Gao N., Yang L., Lu X., Duan Z., Zhu L., Feng J. (2022). A review of interactions of microplastics and typical pollutants from toxicokinetics and toxicodynamics perspective. J. Hazard. Mater..

[73] Wang K., Wang K., Chen Y., Liang S., Zhang Y., Guo C., Wang W., Wang J. (2023). Desorption of sulfamethoxazole from polyamide 6 microplastics: Environmental factors, simulated gastrointestinal fluids, and desorption mechanisms. Ecotoxicol. Environ. Saf..

[74] Adamu H., Haruna A., Zango Z.U., Garba Z.N., Musa S.G., Yahaya S.M., IbrahimTafida U., Bello U., Danmallam U.N., Akinpelu A.A. (2024). Microplastics and Co-pollutants in soil and marine environments: Sorption and desorption dynamics in unveiling invisible danger and key to ecotoxicological risk assessment. Chemosphere.

[75] Zhang R., Li Z., Gao X., Chang S., Yan B., Li G. (2023). Study on Copper Desorption Behavior from Microplastic Particles in Different Media. Water Air Soil Pollut..

[76] Mahendran R., Ramaswamy S.N. (2024). Nanoplastics as Trojan Horses: Deciphering Complex Connections and Environmental Ramifications: A Review. Chem. Afr..

[77] Saud S., Yang A., Jiang Z., Ning D., Fahad S. (2023). New insights in to the environmental behavior and ecological toxicity of microplastics. J. Hazard. Mater. Adv..

[78] Thammatorn W., Palic D. (2022). Potential Risks of Microplastic Fomites to Aquatic Organisms with Special Emphasis on Polyethylene-Microplastic-Glyphosate Exposure Case in Aquacultured Shrimp. Appl. Sci..

[79] Li W., Zu B., Li L., Li J., Li J., Mei X. (2023). Desorption of bisphenol A from microplastics under simulated gastrointestinal conditions. Front. Mar. Sci..

[80] Zhang X., Yu C., Wang P., Yang C. (2025). Microplastics and human health: Unraveling the toxicological pathways and implications for public health. Front. Public Health.

[81] Ioannidis I., Pashalidis I. (2024). Microplastics (PN6) as secondary pollutants: The desorption of radionuclides in simulated human digestive fluids. J. Environ. Chem. Eng..

[82] Patidar K., Alshehri M., Singha W., Alrasheedi M., Younis A.M., Dumka U.C., Ambade B. (2025). Assessing the microplastic pandemic: Prevalence, detection, and human health impacts in Asian aquatic environments. Phys. Chem. Earth.

[83] Zhang F., Man Y.B., Mo W.Y., Man K.Y., Wong M.H. (2020). Direct and indirect effects of microplastics on bivalves, with a focus on edible species: A mini-review. Crit. Rev. Environ. Sci. Technol..

[84] Tien C.-J., Wang Z.-X., Chen C.S. (2020). Microplastics in water, sediment and fish from the Fengshan River system: Relationship to aquatic factors and accumulation of polycyclic aromatic hydrocarbons by fish. Environ. Pollut..

[85] Zhao M., Huang L., Arulmani S.R.B., Yan J., Wu L., Wu T., Zhang H., Xiao T. (2022). Adsorption of Different Pollutants by Using Microplastic with Different Influencing Factors and Mechanisms in Wastewater: A Review. Nanomaterials.

[86] Chen Z., Yang J., Huang D., Wang S., Jiang K., Sun W., Chen Z., Cao Z., Ren Y., Wang Q. (2023). Adsorption behavior of aniline pollutant on polystyrene microplastics. Chemosphere.

[87] Chen L., Shao H., Ren Y., Mao C., Chen K., Wang H., Jing S., Xu C., Xu G. (2024). Investigation of the adsorption behavior and adsorption mechanism of pollutants onto electron beam-aged microplastics. Sci. Total Environ..

[88] Ma J., Zhao J., Zhu Z., Li L., Yu F. (2019). Effect of microplastic size on the adsorption behavior and mechanism of triclosan on polyvinyl chloride. Environ. Pollut..

[89] Zhao H., Hong X., Chai J., Wan B., Zhao K., Han C., Zhang W., Huan H. (2024). Interaction between Microplastics and Pathogens in Subsurface System: What We Know So Far. Water.

[90] Yuan Z., Xu X.-R. (2023). Surface characteristics and biotoxicity of airborne microplastics. Compr. Anal. Chem..

[91] Wang Q., Li T., Tian H., Zou D., Zeng J., Chen S., Xie H., Zhou G. (2024). Effect of pore size distribution of biomass activated carbon adsorbents on the adsorption capacity. J. Chem. Technol. Biotechnol..

[92] Cao X., Wang R., Peng Q., Zhao H., Fan H., Liu H., Liu Q. (2021). Effect of pore structure on the adsorption capacities to different sizes of adsorbates by ferrocene-based conjugated microporous polymers. Polymer.

[93] Bao Z.-Z., Chen Z.-F., Zhong Y., Wang G., Qi Z., Cai Z. (2021). Adsorption of phenanthrene and its monohydroxy derivatives on polyvinyl chloride microplastics in aqueous solution: Model fitting and mechanism analysis. Sci. Total Environ..

[94] Hossain M.D.M., Banerjee A., Chatterjee M., Roy K., Cronin M.T.D. (2024). QSPR and q-RASPR predictions of the adsorption capacity of polyethylene, polypropylene and polystyrene microplastics for various organic pollutants in diverse aqueous environments. Environ. Sci.-Nano.

[95] Du R., Wu W., Ye L., Chen Z., Chen J., Pan Z., Huang X., Luo J. (2025). Plasma-induced aging of microplastics and its effect on mercury transport and transformation. Sep. Purif. Technol..

[96] Hu M., Sun S., Ma H., Xing B. (2025). Adsorption behaviors of microplastics from packaging materials subjected to ultraviolet irradiation and microbial colonization. Mar. Pollut. Bull..

[97] Mao R., Lang M., Yu X., Wu R., Yang X., Guo X. (2020). Aging mechanism of microplastics with UV irradiation and its effects on the adsorption of heavy metals. J. Hazard. Mater..

[98] Zhong Y., Ding Q., Huang Z., Xiao X., Han X., Su Y., Wang D., You J. (2023). Influence of ultraviolet-aging and adsorbed pollutants on toxicological effects of polyvinyl chloride microplastics to zebrafish. Environ. Pollut..

[99] Wu J., Xu P., Chen Q., Ma D., Ge W., Jiang T., Chai C. (2020). Effects of polymer aging on sorption of 2,2′,4,4′-tetrabromodiphenyl ether by polystyrene microplastics. Chemosphere.

[100] Han X.F., Fu L., Yu J., Li K.T., Deng Z.Q., Shu R.H., Wang D.L., You J., Zeng E.Y. (2024). Effects of erythromycin on biofilm formation and resistance mutation of Escherichia coli on pristine and UV-aged polystyrene microplastics. Water Res..

[101] Ding L., Mao R.F., Ma S.R., Guo X.T., Zhu L.Y. (2020). High temperature depended on the ageing mechanism of microplastics under different environmental conditions and its effect on the distribution of organic pollutants. Water Res..

[102] Ma Y., Niu X., Wang X., Min X., Wang X., Guo X. (2025). The sorption behavior of triclosan on microplastics: Aging effects and mechanisms. Chem. Eng. J..

[103] Wang L., Wang S.-X., Zeng X.-Y., He Y., Huang W., Zheng S.-J., Zhang J.-Q. (2022). Effect of Aging on Adsorption of Tetracycline by Microplastics and the Mechanisms. Huan Jing Ke Xue=Huanjing Kexue.

[104] Luo H., Tu C., He D., Zhang A., Sun J., Li J., Xu J., Pan X. (2023). Interactions between microplastics and contaminants: A review focusing on the effect of aging process. Sci. Total Environ..

[105] Tang K.H.D., Li R. (2024). Aged Microplastics and Antibiotic Resistance Genes: A Review of Aging Effects on Their Interactions. Antibiotics.

[106] Titov I., Semerad J., Bohackova J., Benes H., Cajthaml T. (2024). Microplastics meet micropollutants in a central european river stream: Adsorption of pollutants to microplastics under environmentally relevant conditions. Environ. Pollut..

[107] Zhong Y., Wang K., Guo C., Kou Y., Hassan A., Lu Y., Wang J., Wang W. (2022). Competition adsorption of malachite green and rhodamine B on polyethylene and polyvinyl chloride microplastics in aqueous environment. Water Sci. Technol..

[108] Ramesh K., Chellam P.V., Sundaram B. (2024). Transport of layered and spherical microplastics in aqueous ecosystems: A review. Environ. Chem. Lett..

[109] Xu J., Wang L., Sun H. (2021). Adsorption of neutral organic compounds on polar and nonpolar microplastics: Prediction and insight into mechanisms based on pp-LFERs. J. Hazard. Mater..

[110] Goveas L.C. (2025). Interactions between polyaromatic hydrocarbons and microplastics: Environmental mechanisms and ecotoxicological impacts. Environ. Geochem. Health.

[111] Zhang D., Zhang Z., Liu H., Zou J., Yin L., Liu X., Zhang Y.-N., Qu J., Peijnenburg W.J.G.M. (2024). Insights into the effect of crystallinity on the sorption of organic pollutants to microplastics. Environ. Sci. Pollut. Res. Int..

[112] Li C., Liu J., Wang D., Kong L., Wu Y., Zhou X., Jia J., Zhou H., Yan B. (2021). Electrostatic attraction of cationic pollutants by microplastics reduces their joint cytotoxicity. Chemosphere.

[113] Narwal N., Kakakhel M.A., Katyal D., Yadav S., Rose P.K., Rene E.R., Rakib M.R.J., Khoo K.S., Kataria N. (2024). Interactions Between Microplastic and Heavy Metals in the Aquatic Environment: Implications for Toxicity and Mitigation Strategies. Water Air Soil Pollut..

[114] Yu H., Yang B., Waigi M.G., Peng F., Li Z., Hu X. (2020). The effects of functional groups on the sorption of naphthalene on microplastics. Chemosphere.

[115] Tang R., Wen H., Liang J., Zhang X., Sun X., Mai L. (2025). Effects of aging and temperature on the desorption of polychlorinated biphenyls from microplastics in simulated digestive fluids. J. Oceanol. Limnol..

[116] Tang K.H.D. (2021). Interactions of Microplastics with Persistent Organic Pollutants and the Ecotoxicological Effects: A Review. Trop. Aquat. Soil Pollut..

[117] Gu C., Liu W., Zhang Y., Li J., Zhang X., Liu X. (2024). Impact of High Salinity on the Adsorption Behaviors of Polystyrene and Polyamide Microplastics and Alternation of the Toxic Effect toward Synechococcus. Water Air Soil Pollut..

[118] Zhang X., Shen Z., Wu J., Su M., Zheng L., Xie M., Hong H., Huang X., Lu H. (2024). High salinity restrains microplastic transport and increases the risk of pollution in coastal wetlands. Water Res..

[119] Zhang J., Zhang Q., Ma J.P.Y., Shen X., Liang J., Yu L., Ge L., Wang G. (2022). Effects of organic matter on interaction forces between polystyrene microplastics: An experimental study. Sci. Total Environ..

[120] An Q., Zhou T., Wen C., Yan C. (2023). The effects of microplastics on heavy metals bioavailability in soils: A meta-analysis. J. Hazard. Mater..

[121] Xu P., Zhu X., Tian H., Zhao G., Chi Y., Jia B., Zhang J. (2022). The broad application and mechanism of humic acids for treating environmental pollutants: Insights from bibliometric analysis. J. Clean. Prod..

[122] Ali I., Tan X., Li J., Peng C., Naz I., Duan Z., Ruan Y. (2022). Interaction of microplastics and nanoplastics with natural organic matter (NOM) and the impact of NOM on the sorption behavior of anthropogenic contaminants—A critical review. J. Clean. Prod..

[123] Chen Y., Li H., Yin Y., Shan S., Huang T., Tang H. (2023). Effect of microplastics on the adherence of coexisting background organic contaminants to natural organic matter in water. Sci. Total Environ..

[124] Lei J.L., Ma Q.W., Ding X.M., Pang Y.T., Liu Q., Wu J.W., Zhang H.P., Zhang T. (2024). Microplastic environmental behavior and health risk assessment: A review. Environ. Chem. Lett..

[125] Batel A., Borchert F., Reinwald H., Erdinger L., Braunbeck T. (2018). Microplastic accumulation patterns and transfer of benzo[a]pyrene to adult zebrafish (Danio rerio) gills and zebrafish embryos. Environ. Pollut..

[126] Sui Q., Yang X.B., Sun X.M., Zhu L., Zhao X.G., Feng Z.H., Xia B., Qu K.M. (2024). Bioaccumulation of polycyclic aromatic hydrocarbons and their human health risks depend on the characteristics of microplastics in marine organisms of Sanggou Bay, China. J. Hazard. Mater..

[127] Nikhil V.G., Abisha C., Raghavan R., Ali P.H.A., Ranjeet K., Varghese G.K. (2026). Bioaccumulation and trophic transfer of microplastics in oceanic food webs. Mar. Pollut. Bull..

[128] Xu B., Liu F., Cryder Z., Huang D., Lu Z., He Y., Wang H., Lu Z., Brookes P.C., Tang C. (2019). Microplastics in the soil environment: Occurrence, risks, interactions and fate—A review. Crit. Rev. Environ. Sci. Technol..

[129] Li J., Guo K., Cao Y., Wang S., Song Y., Zhang H. (2021). Enhance in mobility of oxytetracycline in a sandy loamy soil caused by the presence of microplastics. Environ. Pollut..

[130] Xie L., Ju J., Pan T., Xiao Y., Zhang T. (2025). Influence mechanism of clay minerals on the adsorption behavior of thiacloprid upon three different acetate microplastics: Effective adsorption sites. Appl. Clay Sci..

[131] Zeng L., Zhou Z., Zhang J., Wang C., Fang C., Ren X., Xiang M., Chen S., Li H. (2024). Selective degradation of organic pollutants in the aquatic environment by microplastic-derived dissolved organic matter through molecular photoresponse sequence transformation. Chem. Eng. J..

[132] Teng X., He M., Xu J., Tang X., Zheng Q., Wang Z., Qu R. (2025). Photochemical transformation and interaction of octachlorodibenzofuran (OCDF) with microplastics in suspended particulate matter-water system. Water Res..

[133] Sabri N.A.A., Razak M.R., Aris A.Z. (2025). Fate of microplastics and emerging contaminants: Mechanisms of interactions, bioaccumulation and combined toxicity to aquatic organisms. Mar. Pollut. Bull..

[134] Chen Y.Y., Cheng X.T., Zeng Y.Q. (2023). The occurrence of microplastic in aquatic environment and toxic effects for organisms. Int. J. Environ. Sci. Technol..

[135] Yang B., Zhou X., Hu X., Qin C., Wang J., Gao Y. (2024). Microplastics may reduce the fish bioaccumulation of organic pollutants. Sci. China Technol. Sci..

[136] Huang W., Song B., Liang J., Niu Q., Zeng G., Shen M., Deng J., Luo Y., Wen X., Zhang Y. (2021). Microplastics and associated contaminants in the aquatic environment: A review on their ecotoxicological effects, trophic transfer, and potential impacts to human health. J. Hazard. Mater..

[137] Xu H., Hu Z., Sun Y., Xu J., Huang L., Yao W., Yu Z., Xie Y. (2024). Microplastics supply contaminants in food chain: Non-negligible threat to health safety. Environ. Geochem. Health.

[138] Chen J., Rao C., Yuan R., Sun D., Guo S., Li L., Yang S., Qian D., Lu R., Cao X. (2022). Long-term exposure to polyethylene microplastics and glyphosate interferes with the behavior, intestinal microbial homeostasis, and metabolites of the common carp (*Cyprinus carpio* L.). Sci. Total Environ..

[139] Rainieri S., Conlledo N., Larsen B.K., Granby K., Barranco A. (2018). Combined effects of microplastics and chemical contaminants on the organ toxicity of zebrafish (Danio rerio). Environ. Res..

[140] Zhang S., Ding J., Razanajatovo R.M., Jiang H., Zou H., Zhu W. (2019). Interactive effects of polystyrene microplastics and roxithromycin on bioaccumulation and biochemical status in the freshwater fish red tilapia (Oreochromis niloticus). Sci. Total Environ..

[141] Han M., Liu H., Zhu T., Tang S., Li Y., Zhu C., Zhou Z., Jiang Q. (2024). Toxic effects of micro(nano)-plastics on terrestrial ecosystems and human health. TrAC. Trends Anal. Chem..

[142] Yang L., Zhang Y., Kang S., Wang Z., Wu C. (2022). Microplastics in soil: A review on methods, occurrence, sources, and potential risk. Sci. Total Environ..

[143] Ullah F., Wang P.-Y., Saqib S., Zhao L., Ashraf M., Khan A., Khan W., Khan A., Chen Y., Xiong Y.-C. (2025). Toxicological complexity of microplastics in terrestrial ecosystems. iScience.

[144] Tang K.H.D., Li R., Li Z., Wang D. (2024). Health risk of human exposure to microplastics: A review. Environ. Chem. Lett..

[145] Emecheta E.E., Pfohl P.M., Wohlleben W., Haase A., Roloff A. (2024). Desorption of Polycyclic Aromatic Hydrocarbons from Microplastics in Human Gastrointestinal Fluid Simulants-Implications for Exposure Assessment. ACS Omega.

[146] Liu L., Tu P., Niu H., Lou X. (2025). Cellular Impact of Micro(nano)plastics on Human Health: A Review. Toxics.

[147] Koelmans A.A., Bakir A., Burton G.A., Janssen C.R. (2016). Microplastic as a Vector for Chemicals in the Aquatic Environment: Critical Review and Model-Supported Reinterpretation of Empirical Studies. Environ. Sci. Technol..

[148] Anilbose K.S., Johnson E., Varghese G.K. (2025). Critical review on microplastics in landfill leachate. Waste Manag. Res..

[149] Hooge A., Hauggaard-Nielsen H., Heinze W.M., Lyngsie G., Ramos T.M., Sandgaard M.H., Vollertsen J., Syberg K. (2023). Fate of microplastics in sewage sludge and in agricultural soils. TrAC. Trends Anal. Chem..

